# The impact of social support on anxiety and depression under the context of COVID-19 pandemic: a systematic review and meta-analysis

**DOI:** 10.3389/fpsyg.2026.1836327

**Published:** 2026-09-14

**Authors:** Meng Wu, Atif Rahman, Eng Gee Lim, Rong Yan

**Affiliations:** 1 Xi’an Eurasia University, Xi’an, China; 2 University of Liverpool, Liverpool, United Kingdom; 3 School of Advanced Technology, Xi’an Jiaotong-Liverpool University, Suzhou, China; 4 Xi’an Jiaotong-Liverpool University, Suzhou, China

**Keywords:** anxiety, COVID-19, depression, meta-analysis, social support, systematic review

## Abstract

**Introduction:**

The COVID-19 pandemic created an extreme and prolonged context of collective stress while simultaneously disrupting ordinary sources of interpersonal support. Although social support is widely associated with better mental health, it remains unclear how consistently this association was observed across populations and contexts during the pandemic and what the findings may imply for future crisis preparedness.

**Methods:**

This systematic review and meta-analysis followed PRISMA 2020 guidelines. Six databases (PubMed, Web of Science, PsycINFO, Taylor & Francis Online, ScienceDirect, and ERIC) were searched for studies published between February 2020 and June 2022. Eligible studies quantitatively examined the association between social support and anxiety and/or depression using validated measures. Study quality was assessed with the Newcastle-Ottawa Scale. Random-effects models were applied to calculate pooled correlations, with subgroup, sensitivity, and publication bias analyses conducted.

**Results:**

Sixty studies comprising 64,693 participants met inclusion criteria. Social support was significantly associated with lower anxiety (ρ = −0.30, 95% CI [−0.33, −0.26]) and lower depression (ρ = −0.33, 95% CI [−0.37, −0.27]). Subgroup analyses showed consistent protective effects across age groups and occupations, particularly among college students and medical workers. Family support demonstrated the strongest associations. Heterogeneity was high (I^2^ > 90%), and publication bias was detected, though trim-and-fill analyses did not alter overall results.

**Conclusion:**

Higher social support was consistently associated with lower anxiety and depression during the COVID-19 pandemic. Viewed as evidence from an extreme and prolonged context of collective stress, these findings may inform mental health preparedness for future public health emergencies and other large-scale crises. However, substantial heterogeneity indicates that the magnitude of the association varies across populations and contexts. Because the pooled estimates were based on bivariate correlations, the findings should not be interpreted as evidence that social support causally was associated with lower symptoms of specific pandemic-related stressors.

## 1 Introduction

Anxiety and depression are the two most prevalent mental health conditions worldwide, contributing significantly to the global burden of disease (World Health Organization [WHO], 2021). During the COVID-19 pandemic, the prevalence of anxiety and depression has sharply increased, particularly among vulnerable groups such as youth and medical workers (Racine et al., 2021; Cao et al., 2020). Anxiety is typically characterized by uncontrollable worry and physiological arousal lasting more than 6 months, while depression is marked by persistent sadness, loss of interest, and functional impairment lasting at least 2 weeks (American Psychiatric Association, 2013). Understanding the risk and protective factors for these conditions is therefore critical.

Among the wide range of psychosocial influences, social support has consistently been identified as a key protective factor (Cohen and Wills, 1985; Rueger et al., 2016). Social support may take the form of emotional, informational, or instrumental resources provided by family, peers, and significant others (Thoits, 2011). However, social support should not be regarded as a single homogeneous construct. Perceived support refers to an individual’s belief that support is available when needed, whereas received support refers to supportive actions that are actually provided. In addition, social embeddedness reflects the extent to which individuals are integrated into social networks and social roles, thereby shaping opportunities to access supportive resources (Berkman et al., 2000). These distinctions are particularly relevant during the COVID-19 pandemic, when physical distancing measures and quarantine restrictions may have altered both the availability and the function of interpersonal support (Zhu et al., 2021).

Several theoretical perspectives help explain how social support may be associated with anxiety and depression. The main-effect model proposes that social support promotes psychological well-being regardless of stress level by enhancing belonging, self-worth, emotional security, and access to practical resources (Cohen and Wills, 1985). In contrast, the stress-buffering model suggests that support protects individuals from the adverse psychological effects of stressful experiences by reducing perceived threat, facilitating coping, and providing emotional or instrumental assistance (Cohen and Wills, 1985). Beyond these perspectives, social support may also operate through dynamic processes. Social mobilization perspectives suggest that stressful events can activate support-seeking behaviors and increase support provision, whereas social deterioration perspectives propose that prolonged stress and psychological distress may gradually erode social relationships and available support resources (Kaniasty and Norris, 2008). Together, these perspectives suggest that the relationship between social support and mental health may be reciprocal, context-dependent, and influenced by both the source and type of support.

Although both the main-effect and stress-buffering models provide important theoretical perspectives for understanding the role of social support, they require different forms of empirical evidence. Evidence consistent with the main-effect model is observed when higher levels of social support are generally associated with better mental health outcomes (van Harmelen et al., 2016). By contrast, demonstrating a stress-buffering mechanism requires studies to directly measure stress exposure and statistically examine whether the association between stress and mental health varies according to the level of social support through a Stress × Support interaction (Cohen and Wills, 1985). Because most studies conducted during the COVID-19 pandemic reported only bivariate associations between social support and psychological outcomes, the present review primarily evaluates evidence consistent with the main-effect framework rather than providing a direct test of the stress-buffering model.

The COVID-19 pandemic represented an extreme and prolonged population-level stress context in which multiple sources of adversity occurred simultaneously. Lockdowns, quarantine measures, school closures, remote working arrangements, concerns about infection, bereavement, and economic uncertainty disrupted ordinary patterns of social interaction and reduced opportunities for face-to-face support (Brooks et al., 2020). At the same time, these conditions increased the need for emotional reassurance, practical assistance, and health-related information, thereby making access to social support particularly salient (Zhu et al., 2021). The pandemic therefore provides more than a temporal backdrop for examining social support. It offers a historically unusual crisis context in which both the demand for support and the barriers to obtaining it were substantially intensified.

Examining social support in this context may provide insights that extend beyond the acute phase of COVID-19. Large-scale public health emergencies, natural disasters, armed conflicts, and other forms of collective disruption may similarly weaken ordinary support systems while increasing psychological distress. Evidence obtained during the pandemic can therefore help clarify whether associations between social support and mental health remain evident under sustained collective stress and whether these associations vary across populations, regions, and sources of support. Such variation is theoretically important because it may indicate that the mental health relevance of social support depends on the social and cultural context in which support is perceived, accessed, and used.

Although numerous empirical studies have examined the association between social support and mental health during the COVID-19 pandemic, the available evidence remains fragmented across populations, regions, and operational definitions of support (Martín-Cano et al., 2022). Existing reviews have often focused on specific groups, such as caregivers or pregnant women, or on particular geographical settings, thereby, limiting broader conclusions (Bedaso et al., 2021; Schiller et al., 2021). Relatively little quantitative synthesis has examined not only the average magnitude of the association between social support and internalizing symptoms but also the extent to which this association varies across different crisis-affected populations and contexts.

This question remains relevant after the end of the acute COVID-19 emergency. If social support is consistently associated with better mental health under conditions of prolonged collective stress, it may represent an important component of preparedness for future crises. Conversely, substantial variation across populations, regions, or sources of support would suggest that support is not a uniformly effective resource and that crisis interventions should be adapted to specific social and cultural contexts. Importantly, however, studies conducted during the pandemic do not provide direct evidence of stress-buffering mechanisms unless they measure pandemic-related stressors and formally test interactions between stress exposure and social support. The present review therefore focuses primarily on the overall association between social support and anxiety and depression during a large-scale crisis context.

The present study conducted a systematic review and meta-analysis of empirical studies examining the association between social support and anxiety and depression during the COVID-19 pandemic. Rather than treating the pandemic solely as a temporal setting, we conceptualized it as an extreme and prolonged context of collective stress in which ordinary social relationships and access to supportive resources were substantially disrupted. The study had three objectives: (a) to estimate the overall magnitude of the associations between social support and anxiety and depression during the pandemic; (b) to examine whether these associations varied across age groups, occupational groups, geographical regions, and sources of support; and (c) to consider what the consistency and variability of these associations may imply for social support theory and preparedness for future large-scale crises. Based on previous theoretical and empirical research, we hypothesized that higher social support would be associated with lower anxiety and depression. Consistent with the available evidence, these hypotheses were framed within the main-effect perspective. Although the stress-buffering model provides an important theoretical explanation for how social support may operate during large-scale crises, the present meta-analysis was not designed to directly evaluate buffering effects because the included studies rarely measured pandemic-specific stressors or tested Stress × Support interactions.

## 2 Methods

### 2.1 Search strategy

A comprehensive literature search was conducted in six electronic databases: PubMed, Web of Science, PsycINFO, ScienceDirect, Taylor & Francis Online, and ERIC. The search covered studies published from February 1, 2020, to June 30, 2022, corresponding to the period of the COVID-19 pandemic.

We combined keywords and MeSH terms related to COVID-19, social support, and mental health outcomes (anxiety, depression) using Boolean operators (AND/OR). The PubMed search strategy was as follows, and similar strategies were adapted for other databases:

An example PubMed search string was as follows:

(“COVID-19” OR “SARS-COV-2” OR “Novel coronavirus” OR “2019-nCov”) AND (“depression” OR “depressive disorder” OR “anxiety” OR “internalizing symptoms” OR “mental health”) AND (“social support” OR “family support” OR “peer support” OR “significant other support”).

Searches were limited to human studies and peer-reviewed journal articles published in English. Reference lists of included studies and relevant reviews were also manually screened to identify additional eligible publications.

### 2.2 Eligibility criteria

The study selection process was conducted and reported according to the PRISMA 2020 guidelines (Page et al., 2021). Studies were included if they met the following criteria: (a) they were empirical quantitative studies published in peer-reviewed journals; (b) they were conducted during the COVID-19 pandemic period; (c) they examined the association between social support and anxiety and/or depression; (d) social support was measured using a validated or clearly described instrument; (e) anxiety and/or depression was assessed using a validated or clearly described measure; (f) sufficient statistical information was available to extract or calculate an effect size; and (g) the study employed a cross-sectional, longitudinal, or diary design.

Studies were excluded if they were qualitative studies, case reports, editorials, conference abstracts, reviews, meta-analyses, intervention studies without relevant baseline associations, studies that did not examine anxiety or depression outcomes, studies that did not assess social support, or studies that lacked sufficient quantitative information for effect-size extraction.

Importantly, studies were not excluded solely because they used online recruitment procedures or convenience sampling methods. Such approaches were widely adopted during the COVID-19 pandemic and were considered eligible when all other inclusion criteria were met. The term “unrepresentative sample population” referred to studies with insufficiently described participant characteristics, highly specialized samples that were not relevant to the review question, or samples for which meaningful interpretation of social support–mental health associations was not possible.

### 2.3 Conceptualization and measurement of social support

In the present review, social support was broadly conceptualized as emotional, informational, instrumental, and relational resources available through interpersonal relationships (Thoits, 1986). However, social support is a multidimensional construct, and the included studies varied in how it was operationalized.

Most studies assessed perceived social support, defined as an individual’s perception that support is available when needed. Common instruments included the Multidimensional Scale of Perceived Social Support (MSPSS; Zimet et al., 1988) and the Perceived Social Support Scale (PSSS), both of which primarily measure perceived support from family, friends, and significant others. Hospital Anxiety and Depression Scale (HADS; Snaith and Zigmond, 1986).

A smaller number of studies employed the Social Support Rating Scale (SSRS), which assesses subjective support, objective support, and support utilization, thereby capturing both perceived and received dimensions of support. Other studies used instruments such as the Medical Outcomes Study Social Support Survey (MOS-SSS), which focuses on functional aspects of support, including emotional, informational, tangible, and affectionate support.

Accordingly, although multiple forms of social support were represented across studies, the evidence synthesized in the present meta-analysis primarily reflects perceived social support, as this was the most frequently assessed construct among the included studies.

### 2.4 Quality assessment

The methodological quality of included studies was assessed using the Newcastle-Ottawa Scale (NOS) for observational studies (Wells et al., 2000). Each study was rated as low, moderate, or high quality across three domains: selection, comparability, and outcome/exposure assessment. Two reviewers conducted the appraisal independently, with disagreements resolved through consensus. Inter-rater reliability was calculated using Cohen’s κ.

### 2.5 Data extraction

A standardized coding form was developed to extract study-level data, including author, year, country, sample characteristics (age, gender, occupation), study design, measurement instruments for social support, measurement instruments for anxiety/depression, and effect size indicators. When multiple effect sizes were reported within the same study, composites were computed to ensure statistical independence (Dahlke and Wiernik, 2019).

The primary effect size was the zero-order Pearson correlation coefficient (r), representing the bivariate association between social support and anxiety or depression. Only zero-order Pearson correlation coefficients (r) were extracted and synthesized in the meta-analysis. Adjusted regression coefficients (β), unstandardized regression coefficients (B or b), and partial correlations were not included in the pooled analyses.

Zero-order Pearson correlation coefficients were selected because they provided a common and directly comparable effect-size metric across studies. Adjusted regression coefficients were not synthesized because the included studies controlled for substantially different sets of covariates, making direct comparison inappropriate. Furthermore, standardized regression coefficients, partial correlations, and other adjusted estimates represent different statistical quantities and therefore cannot be assumed to estimate the same underlying association. Accordingly, the present meta-analysis focused on unadjusted bivariate associations between social support and anxiety or depression.

### 2.6 Statistical analysis

All analyses were conducted in R using the metafor and psychmeta packages. Random-effects models were used because methodological and population-level heterogeneity was expected across studies. Pooled correlations were calculated separately for anxiety and depression.

Heterogeneity was assessed using Cochran’s Q and I^2^ statistics. Subgroup analyses were conducted by age group, occupation, geographical region, type of social support, and analytical role of social support where sufficient data were available. Publication bias was evaluated using funnel plot inspection, Begg’s test, and Egger’s test. Sensitivity analyses were conducted by sequentially removing one study at a time. Results were reported using pooled correlations, 95% confidence intervals, and 80% credibility intervals where applicable.

Meta-regression analyses were conducted as an exploratory approach to investigate potential sources of between-study heterogeneity. Continuous moderators, including mean age, percentage of female participants, and study quality score, were examined separately using random-effects meta-regression models when sufficient data were available. Categorical study characteristics, such as geographical region, study population, and type of social support, were primarily evaluated through subgroup analyses because these variables contained multiple levels and several categories included relatively few studies. Meta-regression results were interpreted cautiously because many study-level characteristics were incompletely reported and substantial residual heterogeneity was anticipated.

## 3 Results

### 3.1 Study selection

A total of 6,662 records were identified through database searches. After removing duplicates and screening abstracts, 424 full-text articles were assessed for eligibility. Sixty studies with a combined sample of 64,693 participants met the inclusion criteria and were included in the final analysis (see Figure 1).

**FIGURE 1:**
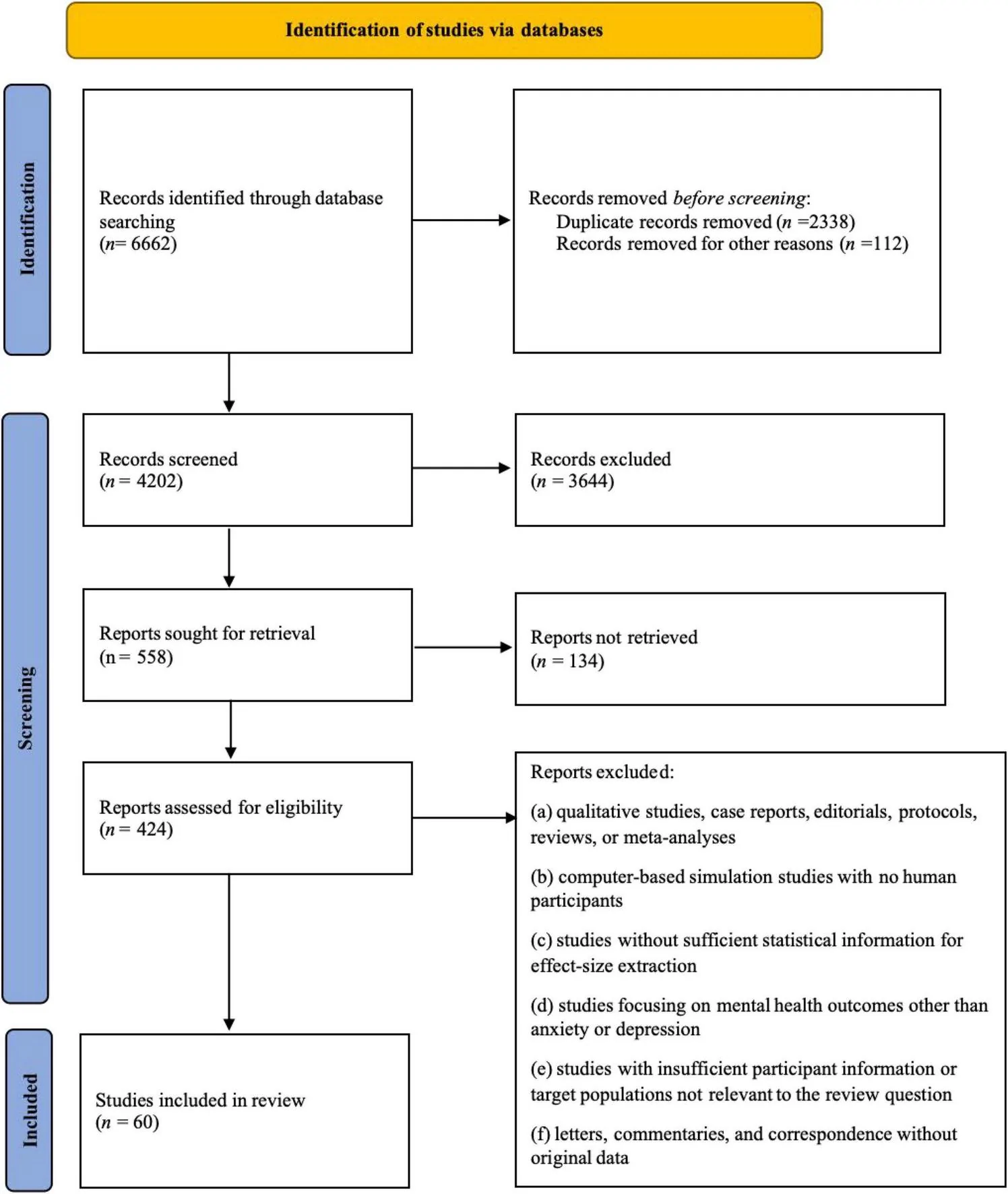
PRISMA 2020 flow diagram of the study selection process.

### 3.2 Study characteristics of included studies

Of the 60 included studies, 53 (88.3%) employed cross-sectional designs and 7 (11.7%) used longitudinal or daily diary designs. Sample sizes ranged from 49 to 7,895 participants. Most studies were conducted in Asia (*n* = 30, 50%) and Europe (*n* = 21, 35%), with fewer in North America (*n* = 7, 11.7%) and South America (*n* = 2, 3.3%). No eligible studies were identified from Africa or Oceania.

Regarding population characteristics, 13 studies targeted children and adolescents (21.7%), 27 studies examined young adults (45.0%), 4 included mid-aged adults (6.7%), and only 2 focused on older adults (3.3%). Occupations represented included the general public (*n* = 15, 25%), college students (*n* = 13, 21.7%), medical workers (*n* = 7, 11.7%), COVID-19 patients (*n* = 2, 3.3%), and pregnant/postnatal women (*n* = 3, 5%).

The most frequently used measures of social support were the Multidimensional Scale of Perceived Social Support (MSPSS), the Perceived Social Support Scale (PSSS), and the Social Support Rating Scale (SSRS). Most included studies assessed perceived social support, whereas a smaller number measured objective support, subjective support, support utilization, or functional support. Therefore, the pooled estimates reported in this review primarily reflect perceived social support rather than actual receipt of support or broader indicators of social embeddedness.

### 3.3 Overall effect of social support on anxiety and depression

The meta-analysis revealed a significant negative association between social support and both anxiety and depression (Table 1; see Tables 2, 3 for subgroup and design-specific analyses).

**TABLE 1:** Characteristics of included studies.

No.	References	Study design	Anxiety measure	Depression measure	*N*	Occupation	Age group	MAge	Female%	Country	Social support measure
1	Feng and Yin, 2021	Cross-sectional	–	CES-D	344	Medical workers	Adults	34.05	65.40%	China	PSSS
2	Alnazly et al., 2021	Cross-sectional	DASS	DASS	365	Medical workers	Adults	NR	55.60%	Jordan	MSPSS
3	Guo et al., 2021	Cross-sectional	DASS	DASS	1278	College students	Adults (young)	NR	64.60%	China	PSSS
4	Kandeğer et al., 2021	Cross-sectional	HADS	HADS	84	Infected patients	Adults (young)	36.71	44.00%	Turkey	MSPSS
5	Harrison et al., 2022a,b	Cross-sectional	PASS	EPDS	205	General public	Adults (young)	NR	100.00%	UK	MSPSS
6	Li et al., 2020	Cross-sectional	GAD-7	PHQ-9	431	College students	Adults (young)	NR	61.90%	China	SSRS
7	Zhuo et al., 2021	Cross-sectional	GAD-7	PHQ-9	1017	College students	Adults	NR	53.30%	China	SSRS
8	Ao et al., 2020	Cross-sectional	STAI		736	General public	Adults (young)	NR	48.30%	China	SSRS
9	Szkody et al., 2021	Cross-sectional	WHOQOL-BREF	WHOQOL-BREF	423	General public	Adults (young)	19.77	65.00%	U.S.	SSRS
10	Shi et al., 2022	Cross-sectional	HADS-A	HADS-D	840	Medical workers	Adults (young)	36.8	79.30%	China	SSRS
11	Shen et al., 2022	Cross-sectional	DASS	DASS	848	Medical workers	Adults (young)	NR	98.70%	China	
12	John-Henderson and Ginty, 2020	Cross-sectional	HADS	HADS	205	General public	Adults (mid-age)	53	59.80%	U.S.	ISEL-12
13	Özmete and Pak, 2020	Cross-sectional	STAI		630	General public	Adults	39.2	73.00%	Turkey	MSPSS
14	Muyor-Rodríguez et al., 2021	Cross-sectional	GAD-7		517	College students	Adults (young)	21.03	20.90%	Spain	MSPSS
15	Li et al., 2021	Cross-sectional	SAS		2640	College students	Adults (young)	NR	68.79%	China	SSQ
16	García-Fernández et al., 2023	Cross-sectional	PRAQ-20		115	Pregnant women	Adults (young)	33.87	100.00%	Spain	MOS-SSS
17	Kang and Kim, 2021	Cross-sectional	K-BSI		316	General public	Adults	43.28	49.40%	South Korea	PSSS
18	Harrison et al., 2022b	Cross-sectional	PASS	EDPS	251	Postnatal women	Adults (young)	NR	NR	UK	MSPSS
19	Shu et al., 2022	Cross-sectional	SAS		2734	College students	Adults (young)	NR	48.20%	China	SSRS
20	Saleh Manijeh et al., 2021	Cross-sectional	CADS		220	Elderly	Adults (senior)	65.7	38.20%	Iran	MSPSS
21	Chen et al., 2021	Cross-sectional	GAD-2		705	Migrant	Adults (young)	NR	47.70%	China	LSNS-6
22	Li and Peng, 2021	Cross-sectional	SAS		2640	College students	Adults (young)	NR	68.79%	China	SSQ
23	Tull et al., 2020	Cross-sectional	SHAI	DASS	500	General public	Adults	40	47.00%	U.S.	MSPSS
24	Çelik et al., 2021	Cross-sectional	BAI		532	General public	Adults	30.95	NR	Turkey	MSPSS
25	Tüzün et al., 2022	Cross-sectional	STAI	BDI	49	College students	Adults (young)	20.53	83.70%	Turkey	MSPSS
26	Li and Peng, 2021	Cross-sectional	STAI		2640	College students	Adults (young)	NR	68.80%	China	CSQ
27	Zhong et al., 2020	Cross-sectional		CES-D	567	Elderly	Adults (senior)	80.5	54.20%	China	MSPSS
28	Amendola et al., 2021	Cross-sectional	BSI	BSI	299	General public	Adults (young)	31.14	NR	Italy	OSSS-3
29	Oryan et al., 2021	Cross-sectional	GAD	PHQ-9	655	General public	Adults	NR	62.60%	Israel	MSPSS
30	Mahamid et al., 2021	Cross-sectional	DASS-21	DASS-21	370	General public	Adults	NR	71.89%	Asia	BSSS
31	Dändliker et al., 2021	Cross-sectional		Two components	1562	Children and adolescents	Children and adolescents	16.18	72%	Switzerland/ Northern/Italy	Previously validated scales
32	Scrimin et al., 2022	Cross-sectional		CHIP-CE	116	Children and adolescents	Children and adolescents	8.67	43.60%	Italy	SCHIP-CE
33	Tucker et al., 2020	Cross-sectional	STSCC		850	Children and adolescents	Children and adolescents	NR	46.34%	U.S.	MSPSS
34	Liu et al., 2020	Cross-sectional		CDI	1512	Children and adolescents	Children and adolescents	15.1	53.80%	China	CASSS
35	Aune et al., 2021	Cross-sectional	SPAIC		7895	Children and adolescents	Children and adolescents	15.92	48.60%	Norway	READ
36	Alshammari et al., 2021	Cross-sectional		CESDC	2741	Children and adolescents	Children and adolescents	NR	NR	Jordan	MSPSS
37	Liu et al., 2022	Cross-sectional		BDI-II	1317	Children and adolescents	Children and adolescents	15.96	53.76%	China	PSSS
38	Ringdal et al., 2020	Cross-sectional	HSCL-10	HSCL-10	1814	Children and adolescents	Children and adolescents	NR	NR	Norway	MSPSS
39	Qi et al., 2020	Cross-sectional	GAD-7	PHQ-9	7202	Children and adolescents	Children and adolescents	16	46.40%	China	SSRS
40	Kurt et al., 2021	Cross-sectional	GAD-7	PHQ-9	345	College students	Adults (young)	33.4	47.80%	Turkey	MSPSS
41	Wang et al., 2022	Cross-sectional	GAD-7	PHQ-9	1573	Earthquake survivors	Adults (young)	26.41	34.50%	China	MSPSS
42	Grey et al., 2020	Cross-sectional	GAD-7	PHQ-9	2020	General public	Adults (young)	NR	NR	Finland	MSPSS
43	Seitz et al., 2021	Cross-sectional	BSI	BSI	85	General public	Adults (young)	31.31	78.80%	Germany	ESSI
44	Basilio et al., 2022	Cross-sectional		DASS-21	160	Children and adolescents	Children and adolescents	NR	NR	NR	VSSS
45	Xiao et al., 2020	Cross-sectional	SAS		180	Medical workers	Adults	NR	71.70%	China	SSSRS
46	Kim et al., 2022	Cross-sectional	GAD-7	CES-D	214	Migrant	Adults (young)	30.1	55.60%	South Korea	MSPSS
47	Hou et al., 2021	Cross-sectional	GAD-7		701	Medical workers	Adults (mid-age)	15.7	93.20%	China	PSSS
48	Reid et al., 2022	Cross-sectional	DASS-21	DASS-21	219	College students	Adults (young)	NR	61.60%	U.S.	MSPSS
49	Corno et al., 2022	Cross-sectional	STAI-6	EPDS	274	Pregnant women	Adults (young)	32.79	100.00%	Canada	MSPSS
50	Długosz et al., 2022	Cross-sectional	GAD-7	PHQ-9	1000	Children and adolescents	Children and adolescents	14.65	50.00%	Poland	SSYSSA-18
			GAD-7	PHQ-9	1022	Children and adolescents	Children and adolescents	13.46	49.00%	Ukrainian	SSYSSA-18
51	Zou et al., 2022	Cross-sectional	GAD-7	PHQ-9	525	Medical workers	Adults	NR	74.70%	China	PPS-5
52	Xu et al., 2022	Cross-sectional	MHI-5	MHI-5	285	Airline pilots	Adults	31.6	0	China	Three-item scale
53	Longest and Kang, 2022	Cross-sectional		CES-D	509	College students	Adults (young)	22.5	NR	U.S.	SSM
54	Kong et al., 2020	Longitudinal	HADS	HADS	144	Infected patients	Adults (mid-age)	49.98	51.39%	China	PSSS
55	Jacmin-Park et al., 2022	Longitudinal	GAD-7	PHQ-9	2908	General public	Adults	NR	NR	UK/France	PSOS
56	Peñate et al., 2020	Longitudinal	GAD-7	PHQ-9	467	Children and adolescents	Children and adolescents	NR	48.82%	Chile	MSPSS
57	Hagiwara et al., 2021	Longitudinal		CES-D	402	Children and adolescents		NR	NR	Japan	RSSS
58	Rodriguez-Rivas et al., 2022	Daily-diary	GAD-7	PHQ-9	444	Children and adolescents	Children and adolescents	15	60%	Chile	NPI
59	Costa et al., 2022	Longitudinal	PRAQ-20		1344	General public	Adults (young)	23.91	67.30%	Italy	O/OSSS
			DASS-21		1344	General public	Adults (young)	23.91	67.30%	Italy	O/OSSS
60	Koutsimani and Montgomery, 2021	Longitudinal	BSI	BSI	76	General public	Adults (mid-age)	40.4	NR	Greece	FSS
			HADS	HADS	55	General public	Adults	39.8	NR	Greece	FSS

NR, Nor reported.

**TABLE 2:** Subgroup analyses by age, occupation, region, and type of social support.

Type of analysis	Groups	*k*	*N*	*−r*	−ρ	SD ρ	95% CI	80% CR	I^2^	*Q*	*P*
Anxiety	Overall effect size	48	52703	−0.26	−0.30	0.14	[−0.333, −0.255]	[−0.469, −0.119]	94.12	850.53^***^	<0.001
Depression	Overall effect size	43	39096	−0.27	−0.33	0.15	[−0.37, −0.28]	[−0.518, −0.132]	95.47	993.98^***^	<0.001
Anxiety	Region										
	Asia	23	29692	−0.27	−0.30	0.13	[−0.367, −0.249]	[−0.483, −0.133]	95.01	440.53^***^	<0.001
	Europe	19	19469	−0.25	−0.28	0.15	[−0.353, −0.208]	[−0.475, −0.086]	95.07	364.89^***^	<0.001
	North America	6	2471	−0.25	−0.29	0.05	[−0.371, −0.215]	[−0.369, −0.217]	48.38	9.69	0.085
	Age										
	Children and adolescents	8	19040	−0.26	−0.31	0.07	[−0.370, −0.253]	[−0.405, −0.218]	90.98	77.58^***^	<0.001
	Young adult	28	25421	−0.27	−0.29	0.14	[−0.343, −0.235]	[−0.467, −0.111]	93.14	393.35^***^	<0.001
	Mid-age adult	4	1126	−0.31	−0.34	0.11	[−0.541, −0.138]	[−0.524, −0.156]	82.51	17.16^***^	0.001
	Whole age-ranged adults	10	6896	−0.23	−0.25	0.23	[−0.418, −0.090]	[−0.567, 0.058]	97.13	313.86^***^	<0.001
	Occupation										
	Children and adolescents students	8	19040	−0.26	−0.31	0.07	[−0.370, −0.253]	[−0.405, −0.218]	90.98	77.58^***^	<0.001
	College students	11	15182	−0.29	−0.33	0.08	[−0.386, −0.274]	[−0.438, −0.222]	80.30	50.75^***^	<0.001
	General public	15	10934	−0.21	−0.23	0.17	[−0.328, −0.137]	[−0.458, −0.007]	94.96	277.67^***^	<0.001
	Medical workers	6	3459	−0.27	−0.30	0.17	[−0.479, −0.121]	[−0.544, −0.056]	93.12	72.67^***^	<0.001
	Pregnant and postnatal women	3	640	−0.21	−0.23	0.13	[−0.604, 0.145]	[−0.479, 0.020]	74.57	7.87*	0.02
	Others	7	3423	−0.23	−0.26	0.32	[−0.562, 0.040]	[−0.725, 0.203]	97.95	292.52^***^	<0.001
	Types of social support										
	Family support	14	9048	−0.25	−0.30	0.07	[−0.345, −0.252]	[−0.390, −0.208]	66.74	39.08^***^	<0.001
	Peer support	10	3444	−0.221	−0.25	0.12	[−0.346, −0.16]	[−0.413, −0.093]	82.64	51.83^***^	<0.001
	Significant others	9	7156	−0.28	−0.30	0.08	[−0.373, −0.236]	[−0.419, −0.19]	84.56	51.81^***^	<0.001
	Role of IV										
	IV	16	29598	−0.27	−0.32	0.10	[−0.377, −0.265]	[−0.458, −0.184]	93.97	248.85^***^	<0.001
	IV with other variables	18	10264	−0.19	−0.22	0.16	[−0.302, −0.139]	[−0.431, −0.011]	92.39	223.31^***^	<0.001
	Mediator/moderator	17	12841	−0.26	−0.30	0.16	[−0.382, −0.211]	[−0.513, −0.079]	94.92	314.87^***^	<0.001
Depression	Region										
	Asia	20	23277	−0.30	−0.35	0.19	[−0.443, −0.262]	[−0.605, −0.099]	97.36	718.21^***^	<0.001
	Europe	17	24614	−0.25	−0.31	0.05	[−0.340, −0.278]	[−0.379, −0.239]	82.98	94.00^***^	<0.001
	North America	7	2980	−0.22	−0.25	0.26	[−0.495, −0.008]	[−0.623, 0.121]	95.98	149.42^***^	<0.001
	Age										
	Children and adolescents	13	32733	−0.28	−0.35	0.09	[−0.406, −0.299]	[−0.469, −0.236]	95.47	265.03^***^	<0.001
	Young adult	20	11056	−0.27	−0.30	0.21	[−0.399, −0.196]	[−0.578, −0.017]	95.71	442.41^***^	<0.001
	Mid-age adult	3	425	−0.24	−0.27	0.16	[−0.728, 0.179]	[−0.572, 0.023]	72.86	7.37*	0.025
	Whole age-ranged adults	8	6599	−0.23	−0.26	0.21	[−0.434, −0.082]	[−0.552, 0.036]	97.11	242.20^***^	0.001
	Occupation										
	Children and adolescents students	14	33135	−0.28	−0.36	0.09	[−0.407, −0.302]	[−0.474, −0.235]	95.24	273.16^***^	<0.001
	College students	7	4520	−0.27	−0.29	0.21	[−0.483, −0.090]	[−0.587, 0.015]	96.52	172.43^***^	<0.001
	General public	10	7376	−0.28	−0.32	0.05	[−0.362, −0.270]	[−0.388, −0.244]	63.28	24.51^***^	0.004
	Medical workers	5	2922	−0.16	−0.17	0.38	[−0.569, 0.227]	[−0.657, 0.315]	97.66	170.67^***^	<0.001
	Other	6	3164	−0.25	−0.29	0.33	[−0.637, 0.064]	[−0.775, 0.202]	98.20	276.99^***^	<0.001
	Types of social support										
	Social support	39	48105	−0.27	−0.34	0.14	[−0.372, −0.280]	[−0.506, −0.146]	95.28	804.41^***^	<0.001
	Family support	15	21454	−0.36	−0.45	0.05	[−0.484, −0.417]	[−0.522, −0.379]	83.40	84.28^***^	<0.001
	Peer support	12	21014	−0.19	−0.24	0.08	[−0.286, −0.183]	[−0.338, −0.131]	90.44	115.05 ^***^	<0.001
	Significant others	7	4946	−0.28	−0.34	0.04	[−0.391, −0.287]	[−0.394, −0.284]	59.39	14.77*	0.022
	Role of IV										
	IV	12	21356	−0.31	−0.35	0.15	[−0.448, −0.260]	[−0.553, −0.155]	97.18	389.83^***^	<0.001
	IV with other variables	18	8387	−0.27	−0.31	0.19	[−0.410, −0.213]	[−0.567, −0.056]	93.37	256.55^***^	<0.001
	Mediator/moderator	16	22039	−0.24	−0.30	0.13	[−0.371, −0.226]	[−0.477, −0.120]	94.91	294.58^***^	<0.001

k, number of effect sizes; *N*, total sample size; $\bar{\text{r}}$, mean observed zero-order Pearson correlation; $\bar{\rho }$, mean corrected correlation; SDρ, standard deviation of corrected correlations; CI, confidence interval; CR, credibility interval; I^2^, percentage of total variability attributable to between-study heterogeneity; Q, Cochran’s heterogeneity statistic; *P*, *p*-value. All effect sizes were based on zero-order Pearson correlations.

*p < 0.05;

**p < 0.01;

***p < 0.001.

**TABLE 3:** Sample size-weighted correlations, true population correlations and 95% confidence intervals of social support in association with anxiety and depression of cross-sectional and longitudinal studies.

Study design	Type of analysis	Groups	*k*	*N*	−*r*	−ρ	SD ρ	95% CI	80% CR	I^2^	*Q*	*P*
Cross-sessional	Anxiety	Overall effect size	47	49504	−0.27	−0.31	0.12	[−0.345, −0.272]	[−0.463, −0.153]	92.67	627.93^***^	<0.001
	Depression	Overall effect size	40	46861	−0.31	−0.37	0.171	[−0.421, −0.309]	[−0.588, −0.142]	96.60	1146.30^***^	<0.001
	Anxiety	Region										
		Asia	23	29918	−0.27	−0.31	0.13	[−0.367, −0.250]	[−0.482, −0.134]	95.00	440.31^***^	<0.001
		Europe	17	16955	−0.28	−0.32	0.10	[−0.369, −0.261]	[−0.447, −0.183]	89.22	148.39^***^	<0.001
		North America	6	2471	−0.26	−0.29	0.05	[−0.371, −0.215]	[−0.369, −0.217]	48.38	9.69	0.085
		Age										
		Children and adolescents	6	19783	−0.28	−0.33	0.06	[−0.404, −0.264]	[−0.428, −0.24]	94.23	86.60^***^	<0.001
		Young adult	27	24077	−0.28	−0.31	0.09	[−0.351, −0.274]	[−0.433, −0.192]	84.53	168.02^***^	<0.001
		Whole age-ranged adults	10	4358	−0.15	−0.17	0.24	[−0.345, 0.012]	[−0.504, 0.170]	95.56	202.47^***^	<0.001
		Occupation										
		Children and adolescents students	6	19783	−0.28	−0.33	0.06	[−0.404, −0.264]	[−0.428, −0.240]	94.23	86.60^***^	<0.001
		College students	12	15527	−0.29	−0.33	0.08	[−0.381, −0.276]	[−0.435, −0.223]	78.67	51.56^***^	<0.001
		General public	14	7068	−0.21	−0.24	0.07	[−0.283, −0.189]	[−0.325, −0.147]	64.19	36.30^***^	0.001
		Infected patients										
		Medical workers	6	3459	−0.27	−0.30	0.17	[−0.479, −0.121]	[−0.544, −0.056]	93.12	72.67^***^	<0.001
		Pregnant and postnatal women	3	640	−0.21	−0.30	0.09	[−0.604, 0.145]	[−0.479, 0.020]	74.57	7.87*	0.02
		Others	5	2867	−0.24	−0.27	0.36	[−0.718, 0.181]	[−0.820, 0.282]	98.60	285.53^***^	<0.001
		Role of social support										
		IV	14	26530	−0.27	−0.32	0.10	[−0.374, −0.259]	[−0.447, −0.187]	92.62	176.25^***^	<0.001
		IV with other variables	16	9823	−0.28	−0.31	0.10	[−0.37, −0.253]	[−0.447, −0.176]	86.11	107.97^***^	<0.001
		Mediator	16	12991	−0.26	−0.30	0.16	[−0.384, −0.207]	[−0.512, −0.079]	95.22	313.88^***^	<0.001
	Depression	Region										
		Asia	19	23101	−0.31	−0.36	0.20	[−0.46, −0.266]	[−0.627, −0.100]	97.78	791.76^***^	<0.001
		Europe	15	21630	−0.31	−0.38	0.10	[−0.441, −0.323]	[−0.521, −0.243]	91.91	173.01^***^	<0.001
		North America	6	2130	−0.22	−0.25	0.30	[−0.569, 0.080]	[−0.694, 0.204]	96.71	152.18^***^	<0.001
		Age										
		Children and adolescents	10	30972	−0.38	−0.42	0.11	[−0.494, −0.339]	[−0.564, −0.269]			
		Young adult	21	12073	−0.27	−0.30	0.20	[−0.394, −0.206]	[−0.568, −0.033]	95.53	447.30^***^	<0.001
		Whole	7	3044	−0.18	−0.21	0.32	[−0.505, 0.090]	[−0.664, 0.248]	97.20	214.14^***^	<0.001
		Occupation										
		Children and adolescents students	10	30972	−0.34	−0.42	0.10	[−0.494, −0.339]	[−0.564, −0.269]	97.23	324.600^***^	<0.001
		College students	8	4865	−0.27	−0.29	0.20	[−0.458, −0.117]	[−0.570, −0.005]	95.95	172.667^***^	<0.001
		General public	10	4854	−0.29	−0.33	0.05	[−0.381, −0.282]	[−0.402, −0.261]	58.35	21.606*	0.01
		Medical workers	5	2922	−0.16	−0.17	0.32	[−0.569, 0.227]	[−0.657, 0.315]	97.66	170.668 ^***^	<0.001
		Other	7	3248	−0.26	−0.30	0.32	[−0.591, 0.012]	[−0.754, 0.175]	97.86	279.739 ^***^	<0.001
		Role of social support										
		IV	11	18448	−0.33	−0.38	0.17	[−0.499, −0.270]	[−0.615, −0.153]	97.81	456.445^***^	<0.001
		IV with other variables	15	7074	−0.27	−0.31	0.20	[−0.418, −0.191]	[−0.572, −0.037]	94.25	243.343^***^	<0.001
		Mediator/moderator	14	21339	−0.30	−0.37	0.17	[−0.468, −0.272]	[−0.596, −0.144]	96.91	420.692 ^***^	<0.001
Longitudinal	Anxiety	Overall effect size	6	5383	−0.21	−0.24	0.10	[−0.484, 0.011]	[−0.580, 0.107]	97.88	235.624^***^	<0.001
	Depression	Overall effect size	6	4441	−0.28	−0.31	0.08	[−0.400, −0.218]	[−0.424, −0.194]	67.07	15.182*	0.010

Mean r, sample size-weighted correlations; mean ρ, true population correlations; CI, 95% confidence intervals; CV, 80% credibility intervals; k, numbers of studies; *N*, total number of participants; SD ρ, estimated standard deviation of the true population correlation; Q, Chi-square test of homogeneity; I^2^, the proportion of true variance to total variance. Subtypes that were less than three cases were not reported.

*p < 0.05;

**p < 0.01;

***p < 0.001.

For anxiety, the pooled sample-size weighted correlation was *r* = −0.26, 95% CI [−0.33, −0.26], *p* < 0.001, based on 48 studies (*N* = 52,703). The true population correlation (ρ) was estimated at −0.30. Heterogeneity was high, Q(47) = 850.53, *p* < 0.001, I^2^ = 94.1%.

For depression, the pooled correlation was *r* = −0.27, 95% CI [−0.37, −0.28], *p* < 0.001, based on 43 studies (*N* = 39,096). The estimated true population correlation was ρ = −0.33, with significant heterogeneity, Q(42) = 993.98, *p* < 0.001, I^2^ = 95.5%.

These results indicate that higher levels of social support were consistently associated with lower levels of both anxiety and depression across the included studies.

### 3.4 Subgroup analyses

#### 3.4.1 By age

Negative associations were significant across children/ adolescents (*r* = −0.26, 95% CI [−0.37, −0.25]), young adults (*r* = −0.27, 95% CI [−0.34, −0.24]), and mid-aged adults (*r* = −0.31, 95% CI [−0.54, −0.14]). The effect was weaker and non-significant among older adults, though only two studies were available.

#### 3.4.2 By occupation

Associations were strongest among college students (*r* = −0.29, 95% CI [−0.39, −0.27]) and children/adolescents (*r* = −0.28, 95% CI [−0.37, −0.25]). Moderate effects were observed for medical workers (*r* = −0.27, 95% CI [−0.48, −0.12]) and pregnant/postnatal women (*r* = −0.21, 95% CI [−0.60, 0.15], ns).

#### 3.4.3 By region

Significant negative associations were found in studies conducted in Asia (*r* = −0.27, 95% CI [−0.37, −0.25]) and Europe (*r* = −0.25, 95% CI [−0.35, −0.21]). Studies from North America also showed a negative association between social support and anxiety. (*r* = −0.25, 95% CI [−0.37, −0.21]). However, the estimate was based on only six effect sizes. Within-subgroup heterogeneity was not statistically significant, Q(5) = 9.69, *p* = 0.085, I^2^ = 48.4%.

#### 3.4.4 By type of social support

Subgroup analyses indicated that family support (*r* = −0.25, 95% CI [−0.35, −0.25]), peer support (*r* = −0.22, 95% CI [−0.35, −0.16]), and support from significant others (*r* = −0.28, 95% CI [−0.37, −0.24]) were all significantly associated with lower anxiety and depression.

### 3.5 Meta-regression analyses

Exploratory random-effects meta-regression analyses were conducted to examine whether selected continuous study characteristics contributed to the substantial between-study heterogeneity. Mean age, percentage of female participants, and study quality score were evaluated separately for anxiety and depression. None of these continuous moderators significantly explained the variability in effect sizes across studies (all *p*s > 0.05).

### 3.6 Sensitivity and publication bias analyses

Sensitivity analyses, conducted by sequentially removing one study at a time, did not substantially alter the overall results, indicating robustness of the findings.

Begg’s and Egger’s tests indicated potential publication bias for both anxiety (Egger’s test, *p* = 0.04) and depression (*p* = 0.03). Funnel plot inspection suggested slight asymmetry, with smaller studies reporting stronger negative associations. Trim-and-fill analyses, however, did not significantly alter the pooled estimates.

## 4 Discussion

### 4.1 Summary of main findings

The present systematic review and meta-analysis synthesized evidence from 60 studies involving 64,693 participants and found that higher social support was associated with lower anxiety and depression during the COVID-19 pandemic. Although the acute public health emergency has ended, these findings remain relevant because the pandemic represented an extreme and prolonged context of collective stress in which ordinary support systems were disrupted at the same time that the need for support increased.

From this perspective, the findings contribute more than a retrospective description of mental health during COVID-19. They indicate that the inverse association between social support and internalizing symptoms remained evident across a wide range of populations during a period of sustained uncertainty, social restriction, health threat, and economic disruption. At the same time, the substantial variation across studies suggests that the association was not uniform. The psychological relevance of social support may depend on the population, social context, cultural meaning of support, and source from which support is obtained.

The present meta-analysis demonstrated that higher social support was consistently associated with lower levels of anxiety and depression during the COVID-19 pandemic. This pattern is broadly consistent with the main-effect model of social support, which proposes that supportive relationships are generally associated with better psychological well-being regardless of the level of stress exposure (Cohen and Wills, 1985). However, consistency with the main-effect framework should not be interpreted as direct confirmation of the model because the present review synthesized observational associations rather than experimentally manipulated social support.

Importantly, the present findings should not be interpreted as evidence supporting the stress-buffering model. A buffering effect requires empirical demonstration that social support moderates the association between stress exposure and psychological outcomes. Most included studies did not directly measure pandemic-related stressors, such as infection exposure, quarantine experiences, bereavement, financial loss, or perceived COVID-19-related stress, nor did they statistically evaluate Stress × Support interaction effects. Consequently, the current meta-analysis cannot determine whether social support attenuated the psychological consequences of specific pandemic-related stressors.

### 4.2 Interpretation of main findings

The overall pooled correlations indicated moderate negative associations between social support and both anxiety (*r* = −0.26) and depression (*r* = −0.27). These results suggest that social support functioned showed a robust inverse association with anxiety and depression. Subgroup analyses revealed that this association was evident across children, adolescents, young adults, and medical workers, although the magnitude varied. Consistent with prior evidence (Gariepy et al., 2016), family support appeared particularly salient, underscoring its central role as a protective correlate of psychological distress.

Negative associations were also observed in North American samples, although the small number of effect sizes limits firm regional comparisons. This divergence may reflect cultural differences in the perception and utilization of social support (Taylor et al., 2004), methodological variation, or the limited number of studies conducted in this region. Similarly, weaker associations among older adults may reflect under-representation of this group, challenges in recruitment during pandemic restrictions, or reliance on institutional rather than familial support networks.

### 4.3 Heterogeneity and sources of variation

The substantial heterogeneity observed across studies should be regarded as an important finding rather than merely a statistical limitation. Although random-effects models account for variation in true effect sizes, the high I^2^ values indicate that the association between social support and mental health differed considerably across populations and study contexts. Consequently, the pooled correlations should be interpreted as average associations rather than universal effect sizes applicable to all settings.

Exploratory meta-regression analyses indicated that commonly reported study characteristics, including mean age, percentage of female participants, and study quality, did not significantly account for the observed heterogeneity. This finding suggests that demographic characteristics alone are unlikely to explain why the association between social support and mental health varies across studies.

By contrast, subgroup analyses indicated that effect sizes differed across populations and sources of social support. For example, family support tended to show stronger associations than peer support for depression, whereas the magnitude of the association also varied across occupational and regional groups. Although these findings should be interpreted cautiously, they suggest that social support operates within broader social and cultural contexts rather than functioning as a universally equivalent resource.

Importantly, substantial residual heterogeneity remained after moderator analyses. This suggests that additional contextual factors, including cultural norms, accessibility of support, pandemic severity, public health restrictions, digital communication, and socioeconomic conditions, may influence the association between social support and mental health but were not consistently reported across the included studies. Future research should therefore move beyond demographic characteristics and examine how contextual factors shape the effectiveness and availability of social support during large-scale crises.

### 4.4 Implications for crisis preparedness and mental health practice

The findings have implications for preparedness for future public health emergencies and other forms of large-scale social disruption. Crisis-response planning should treat the maintenance of supportive relationships as an important component of mental health preparedness, particularly when physical distancing, displacement, institutional closure, or disruption of community services limits ordinary forms of interpersonal contact.

However, the high between-study heterogeneity indicates that a universal support strategy is unlikely to be equally appropriate for all populations. Support initiatives should be tailored to the needs, social networks, and cultural contexts of specific groups. For example, family-based support may be especially relevant in some populations, whereas peer, professional, community, or digitally mediated support may be more accessible or acceptable in others. Healthcare workers, young people, pregnant and postnatal women, and socially isolated individuals may also face different barriers to seeking and receiving support.

The findings further suggest that crisis-preparedness systems should consider not only whether support is available but also whether it is perceived as reliable, accessible, and responsive to individuals’ needs. Community-based programs, peer-support networks, family-focused services, and digital communication platforms may help preserve social connection when face-to-face contact is restricted. Nevertheless, because the evidence synthesized in this review was predominantly correlational, these implications should be regarded as directions for intervention development and evaluation rather than as proof that increasing support will directly reduce anxiety or depression.

### 4.5 Strengths and limitations

This review has several strengths, including adherence to PRISMA guidelines, the inclusion of a large and diverse sample, and the use of rigorous quality assessment procedures. The integration of both cross-sectional and longitudinal evidence allowed for more comprehensive conclusions.

Some limitations should be noted. First, most included studies used cross-sectional designs, which limits causal interpretation. Second, variation in measurement instruments may have contributed to heterogeneity across studies. Third, many studies used online recruitment or convenience sampling, which was common during the COVID-19 pandemic but may affect generalizability.

In addition, because all pooled effect sizes were based on bivariate correlations, the findings should be interpreted as evidence of associations between social support and mental health outcomes during the pandemic rather than as evidence that social support mitigated the effects of specific COVID-19-related stressors.

Another limitation relates to the effect-size metric used in the present meta-analysis. To maximize comparability across studies, only zero-order Pearson correlations were synthesized. Consequently, the pooled estimates represent unadjusted associations between social support and anxiety or depression rather than the independent association after accounting for potential confounding variables. Factors such as age, socioeconomic status, pre-existing mental health conditions, physical health, living arrangements, and exposure to pandemic-related stressors may influence both perceived social support and psychological outcomes. Therefore, the observed correlations should not be interpreted as estimates of the unique contribution of social support.

### 4.6 Directions for future research

Future research should move beyond estimating overall associations between social support and mental health by directly testing the theoretical mechanisms through which social support may influence psychological outcomes. In particular, longitudinal and experimental studies should simultaneously assess pandemic- or crisis-related stressors and examine Stress × Support interaction effects to determine whether social support functions primarily through a main-effect process, a stress-buffering mechanism, or a combination of both. Such designs would also help distinguish whether social support primarily promotes psychological well-being regardless of stress exposure or whether it becomes particularly beneficial under conditions of elevated stress. Future research should use longitudinal and experimental designs to clarify the causal pathways between social support and mental health. Greater attention should also be given to underrepresented populations, including older adults and individuals in low- and middle-income countries. To reduce heterogeneity and improve comparability, future studies should adopt standardized measures of social support. Researchers should also carefully assess pandemic-related stressors, such as infection exposure, quarantine experiences, bereavement, and perceived pandemic-related stress, and directly examine how social support shapes mental health outcomes in these contexts. Further work is needed to investigate digital and online forms of social support, as these may have distinct implications during and after periods of pandemic-related social restriction.

## 5 Conclusion

This systematic review and meta-analysis found that higher social support was consistently associated with lower anxiety and depression during the COVID-19 pandemic. By examining these associations within an extreme and prolonged context of collective stress, the review provides insights that extend beyond the acute pandemic period. The findings suggest that preserving access to meaningful supportive relationships may be an important consideration in mental health preparedness for future public health emergencies and other large-scale crises.

At the same time, the substantial heterogeneity across studies indicates that the association between social support and mental health is context- and population-dependent rather than uniform. Crisis-response strategies should therefore be tailored to the needs of specific populations and should consider the source, accessibility, and cultural meaning of support. Because the pooled estimates were based primarily on bivariate correlations, the findings do not establish that social support caused reductions in anxiety or depression or buffered the effects of specific pandemic-related stressors. Rather than indicating a single universal effect, the findings suggest that the mental health relevance of social support is context-dependent. Understanding the conditions under which different forms of support are most beneficial may therefore represent an important direction for future research and crisis preparedness.

## Data Availability

The original contributions presented in the study are included in the article/supplementary material, further inquiries can be directed to the corresponding author.
